# 

**Published:** 1885-02

**Authors:** 


CONTENTS.

Vol. 32. No. 2.

Disinfectants ..... 1
Pure Water ..... 2
Water Poultices ..... 3
Why People take Medicine ..... 4
Precaution against Cholera ..... 5
Hot Water for inflamed mucous surface ..... 6
Source of the Sun's heat ..... 6
The art of early rising ..... 7
Paper from Wood ..... 8
Fruits as Food and Medicine ..... 9
The rigidity of the Earth ..... 10
Obituary ...... E. H. Gibbs, A. M., M. D ..... 11
Quinine and the hearing ..... 12
On the Mississippi ..... 12
Mirror Lake ..... 13
Sacro-Peptones ..... 14
Danger of bathing when heated ..... 15
How a needle travels ..... 15
Coffee as a stimulant ..... 16
Measuring the heighth of a tree ..... 17
Notes and Miscellany ..... 18, 19, 20

